# Sports Participation, Social Networks, and Sexual Violence Perpetration

**DOI:** 10.1177/08862605221092067

**Published:** 2022-04-26

**Authors:** Kyle K. Nickodem, Kathleen C. Basile, Dorothy L. Espelage, Ruth W. Leemis, Katherine M. Ingram, Colleen Barbero

**Affiliations:** 1 2331 University of North Carolina at Chapel Hill, USA; 2 Division of Violence Prevention, National Center for Injury Prevention and Control, 1242 Centers for Disease Control and Prevention, Atlanta, GA, USA

**Keywords:** sexual harassment, sexual violence, adolescents, sports participation, social networks

## Abstract

Adolescent sexual violence (SV), which includes non-contact verbal sexual harassment (SH) and forced sexual contact (FSC), is a significant public health problem with long-term impacts on health and well-being. Understanding how sports participation is linked to SV can inform prevention efforts; however, the current literature is unclear about the nature of this association. Using data from 20 high schools, we investigate whether athletes in certain sports are at higher risk of SH and FSC perpetration than either other athletes or sports non-participants, and whether the risk is moderated by gender, dismissiveness of SV, or substance use intentions. We also utilize social network data to explore the role of relationships with peers and trusted adults to attenuate SH and FSC perpetration. Second, we incorporate characteristics of friends to further examine the role and composition of peer groups in the association between sports participation and perpetration of SH and FSC. Findings revealed a bivariate association between sport contact level and SH perpetration, but not FSC, and the association disappeared after adjusting for other covariates. Most prominently, dismissiveness of SV, intentions to use substances, and prior perpetration had the strongest association with perpetration regardless of sport contact level. Results also provided some support for the influence of peers and trusted adults in the sports context. Notably, the percentage of friends who perpetrated FSC and the percentage of friends who play a low-contact sport were positively associated with FSC perpetration, and the percentage of friends who play a high-contact sport was positively associated with SH perpetration. The paper concludes with a discussion of the sports context as an important venue for comprehensive prevention efforts, including a focus on changing norms around adolescent SV and substance use.

Broadly defined as any unwanted sexual behavior along a spectrum from non-contact verbal sexual harassment to rape (Basile et al., 2014), sexual violence (SV) is a significant public health problem that often emerges in adolescence and has long-term impacts on health and well-being (Smith & Breiding, 2011). Understanding how sports participation may be linked to SV is relevant to prevention efforts; however, the current literature is unclear about the nature of this association. While a growing body of research finds that athletes are more likely than non-athletes to perpetrate SV (Basile et al., 2020; Gage, 2008), there is also literature connecting sports involvement with risk and protective factors associated with SV (Eime et al., 2013). Research suggests both sports participation and SV perpetration are influenced by peer relationships, but few studies have examined sports, SV perpetration, and peer relationships together (Kreager, 2007). Until recently, most scholarship linking sports participation to SV perpetration has focused on college-aged men (Raliance, 2017). Thus, a better understanding of the role(s) of sports in SV perpetration may inform the primary prevention of SV perpetration, particularly in adolescence when SV perpetration emerges (Ybarra & Thompson, 2018) and sports participation increases (Logan & Cuff, 2019).

## Sports Participation as Risk Factor

### Types of Sports

Characteristics of sports have been explored in attempts to explicate the sports participation-SV perpetration association (Gage, 2008). Higher rates of SV perpetration have been found for adolescent boys participating in high contact sports compared to boys who do not participate in sports, and adolescent girls participating in non- or low-contact sports compared to girls not participating in sports (Basile et al., 2020). Literature and theory might help explain the contact team sports and SV perpetration link. For example, team sports often connote strength, power, and masculinity, which sanctions the use of aggression (Edley & Wetherell, 1997). In a study of both male and female athletes aged 15–39, the athletes in high contact sports reported more aggressive behaviors than athletes in low contact sports (Sofia & Cruz, 2017). The sports participation-SV link is also demonstrated through the pervasive use of violent language to describe winning a competition (e.g., “raping” the opponent), objectification of women, and using queerness to signify weakness (Adams et al., 2010; Reigeluth & Addis, 2021; Young et al., 2017). This atmosphere of violence suggests athletes, particularly male athletes, on contact sport teams would be more likely than other athletes or non-athletes to perpetrate SV, which is supported in the few existing studies examining sport contact level (Basile et al., 2020; Ingram et al., 2022). Less is known about girls' and non-binary genders’ sports involvement and SV perpetration, and theoretical frameworks to explain it. Studies speculate that girls’ participation in sports may situate them where SV perpetration and victimization are more likely (Basile et al., 2020; Bjørnseth & Szabo, 2018).

### Attitudes and Behaviors

Literature suggests SV perpetration is more common among those who hold more general pro-violence attitudes and beliefs (e.g., hostility towards women, traditional ideas about masculinity; Reidy et al., 2015; Tharp et al., 2013). The culture of sports teams may play a role in shaping those beliefs. A meta-analysis of 27 studies found the moral climate—the shared norms and values—of a sports team is associated (*r =* .40) with the prosocial and antisocial behaviors of athletes on the team (Spruit et al., 2019). Among undergraduate men, Young and colleagues (2017) found recreational and intercollegiate athletes were more accepting of rape myths and had greater adherence to traditional gender roles than non-athletes. Furthermore, research suggests that substance use, particularly alcohol, is associated with SV perpetration (Basile et al., 2018; Tharp et al., 2013) and sports participation (Kwan et al., 2014). Ingram et al. 2022 found that U. S. high school athletes who engaged in more than one type of substance use were more likely than their non-substance using peer-athletes to report perpetrating SV, regardless of the type of sport.

### Peer Relationships

Decades of research and theory have implicated peer relationships in explaining male violence against others, and especially against women (DeKeseredy & Schwartz, 1993; 2013; Schwartz & DeKeseredy, 1997). The male peer support (MPS) model (DeKeseredy, 1988), which suggests that attachments to male peers and their resources (e.g., support) legitimizes and encourages abuse of women, has expanded to incorporate other factors, such as substance use and membership in male groups (e.g., college fraternities, sports teams; Schwartz & DeKeseredy, 1997). Recent scholarship has emphasized the role sports might play in understanding forms of masculinity and violence against women (Schwartz, 2021). DeKeseredy and colleagues (2017) recognized that MPS theory can apply to other forms of aggression (e.g., male on male, female on male) and have renamed MPS to be pro-abuse peer support.

In addition to peer support theory, recent scholarship has shown that aspects of social networks, such as position in the network and quality of friendships, can influence health and aggression (Sijtsema & Lindenberg, 2018; Tucker et al., 2016). For example, higher network centrality has been associated with aggressive behavior during adolescence (Rodkin et al., 2006; Sijtsema & Lindenberg, 2018). However, there is an absence of literature to help understand how social networks may affect the perpetration of SV in the context of adolescent sports.

## Sports Participation as Protective Factor

### Well-Being

Theoretical models that conceptualize violent behavior (broadly, not just SV) as a perceived means by which to meet one’s needs posit that holistic well-being is a primary buffer against violent behavior (Guerra, 2003). While this specific pathway has not been empirically examined in the context of sports preventing SV, research supports the components of the pathway. Positive psychosocial and interpersonal factors, such as emotional well-being and social support, have been connected to lower risk for SV perpetration (Basile et al., 2018; Tharp et al., 2013). In a systematic review of the benefits of sports among children and adolescents, sports involvement was associated with better emotional well-being and regulation, higher self-esteem and confidence, and higher relationship quality (Eime et al., 2013). Team sports involvement was found to be particularly beneficial, as it has been associated with lower risk-taking, mental or general health problems, depressive symptoms, and social isolation, as well as greater life satisfaction and social acceptance than non-participation (Eime et al., 2013). One longitudinal study of adolescent girls found that team sport achievement was associated with higher self-esteem 3 years later (Pedersen & Siedman, 2004).

### Peer Relationships

Another mechanism by which sports may prevent individuals from engaging in SV is through healthy peer relationships. Several studies have documented that sports participation offers students opportunities to form close, positive peer relationships, and practice interpersonal communication in the context of working together as a team (Bedard et al., 2020; Eime et al., 2013). Social network diversity may also be protective against SV, especially among boys. Adolescents in schools with frequent boy-girl interactions were associated with diminished aggression (Faris & Felmlee, 2011). Additionally, research shows that college-aged men with greater social network diversity tend to commit fewer acts of SV (Kaczkowski et al., 2017). In men aged 18–29 years old, those with more diverse networks were also less likely to hold beliefs similar to their peers, including attitudes related to violent extremism (Kaczkowski et al., 2020). However, sports as an opportunity for adolescents to build more diverse social networks to protect against SV has not been examined.

### Trusted Adults

Adolescence is a developmental stage when youth often form relationships with non-parental adults. Most adolescents report having trusted adults in their lives that serve different roles than friends or parents (Beam et al., 2002). Evidence suggests that the presence of trusted adults is associated with lower levels of behavioral problems, including misconduct, substance abuse, and sexual activity (Sterrett et al., 2011). With a focus on sexual harassment (SH) among adolescents, one latent class analysis found that having caring adults in the school community was a direct protective factor against being in the SH victimization and perpetration class (Doty et al., 2017).

### Present Study

The collective literature indicates that sports participation may serve as a possible mechanism protecting against SV by promoting individual (e.g., greater emotional health) and relational benefits (e.g., more social connectedness); however, sports may also perpetuate norms (e.g., pro-violence attitudes, substance use) that increase the risk of SV perpetration. The purpose of the present study is to examine sports participation as a risk and/or protective factor for two types of SV perpetration—SH and forced sexual contact (FSC)—to inform prevention among adolescents. SH is defined as non-contact unwanted sexual experiences taking the form of written or verbal comments or creating a sexually hostile climate in person or via technology (Basile et al., 2014). FSC is defined as unwanted, intentional sexual touching either perpetrated against a victim or by forcing a victim to touch the perpetrator. These two types of SV were chosen given their prevalence in adolescents (Ybarra & Thompson, 2018).

In the first analysis, using the full sample of students, we investigate the research questions: (1) compared to students who do not participate in sports, is participation in high-, low-, or no-contact sports differentially associated with SH and FSC perpetration; (2) to what extent are the associations between perpetration and sports participation moderated by gender, dismissive attitudes toward SV, or substance use intentions; and (3) to what extent are type and total number of trusted adults reported by a student associated with SH or FSC perpetration? Additionally, we utilize social network data to explore the association of students’ social connectedness with peers on SH and FSC perpetration. In a second analysis of students who nominated friends, we incorporate characteristics of friends (e.g., percent of friends of the same gender, percent of friends who play a high-contact sport) to further examine the role and composition of peer groups in the association of sports participation with SH and FSC perpetration.

## Methods

### Participants

Data for the present study came from the final wave (Wave 4) of a longitudinal randomized controlled trial investigating the efficacy of an intervention for youth wellness promotion and suicide prevention. The intervention had no effect on SV perpetration rates (Espelage et al., in prep); therefore, participants from both arms of the trial were included in this study. Surveys were collected from 3506 students at 20 Colorado high schools in Spring 2019. Participants were only asked about sports participation at Wave 4. Thus, the association between sports participation and perpetration of SH and FSC in Wave 4 was cross-sectional and observational, albeit with the advantage of utilizing prior reports of SH and FSC perpetration during Waves 1–3 (Fall 2017-Fall 2018) as control variables in the analyses. The racial and ethnic composition of the sample was 46% Hispanic, 38% White, 10% Multiracial, 3% Asian or Pacific Islander, 2% Black, and 1% Indigenous. In the sample, 47% of students identified as female, 51% as male, and 2.5% as other gender. Additionally, 3.2% of students reported as transgender and 22% reported as a sexual minority (see Demographic section for coding decisions). The average age of participants was 16.4 years (*SD* = 0.96). The sample for the second analysis included the 1665 students (52.5%) who named a friend (defined in the Friendship Networks section) via the self-report survey.

### Procedures

The study and a waiver of active parental consent was approved by four institutional review boards (IRBs). Parents could opt their child out of the study by calling the school or researcher or by returning a signed parent form. Non-consented youth did not complete the survey and were removed from the room during survey administration. Students provided assent to participate by signing the front page of the survey. A 68.5% participation rate was achieved in the study schools in Wave 4.

Two research assistants administered the survey in high school classrooms with IRB requirements that teachers not be present. At the beginning of each survey administration, students were told about the purpose of the project and their rights related to participation. Students could skip questions they did not want to answer and were also told they could stop responding at any point. Students were given resources for violence victimization, mental health issues, or suicidal behaviors at the end of the survey.

### Measures

#### Outcomes: Sexual Harassment and Forced Sexual Contact Perpetration

Sexual harassment and FSC perpetration were measured using 13-items from a modified version of the American Association of University Women Sexual Harassment Survey—Perpetration Scale (Espelage et al., 2015). On this scale, students indicated how often in the past 6 months they engaged in unwanted sexual behaviors toward other students at school. Response options ranged from 0 (*Never*) to 4 (*7 or more times*). The first 4 items measured non-contact verbal SH perpetration, including “made sexual comments, jokes, gestures, or looks” and “spread sexual rumors about them” (α = .81, ω_h_ = .85). The remaining eight items measured FSC perpetration varying in severity from “touched, grabbed, or pinched in a sexual way” to “forced them to have sex when they did not want to” (α = .97, ω_h_ = 1.00). Due to low frequencies on the upper end of the response scale and to improve model fit, dichotomous SH and FSC variables were created as the analysis outcomes. A student was coded as 1 (*perpetrator*) if they gave a response of 1 or higher to any item in the scale and 0 (*non-perpetrator*) if a response of 0 was given for all items.

#### Prior SH and FSC Perpetration

Using the same procedures as the outcomes, dichotomous variables for prior SH and FSC perpetration were created based on responses to the items in Waves 1–3 (Fall 2017-Fall 2018). Across the three waves, reliability for SH perpetration was between α = .59–.75 (ω_h_ = .67–.81) and between α = .90–.97 (ω_h_ = .95–1.00) for FSC perpetration.

#### Sports Participation

Students were asked “Did you play on a sports team that was affiliated with your school this school year?” Students could report up to three sports they played from a drop-down list of 30 sports, including an option for “no sports participation.” Each sport was categorized as a non-contact, low-contact, or high-contact sport based on policy statements made by the American Academy of Pediatrics Committee on Sports Medicine and Fitness (2001). Students were then categorized by the highest contact level in which they participated. This created four mutually exclusive categories for sports participation (no sports, non-, low-, and high-contact).

#### Demographics

Each student was asked to report gender, sexual orientation, and race/ethnicity at each of the four waves. For these questions, students could check all identities that applied. A student was coded as transgender if they identified as transgender in any wave. Gender identity was coded separately from transgender status with students who responded as other gender in any of the four waves and students who responded as both male and female equally across the waves coded as other gender. For students who selected multiple sexual orientations, priority was given to responses of other sexual orientation, questioning/unsure, gay/lesbian, and bisexual over responses of heterosexual. Nonetheless, these groups were combined as sexual minority due to low frequencies. A student’s mode response across the four waves was used as their racial/ethnic category in the analyses. Due to low frequencies, Multiracial, Asian or Pacific Islander, Black, and Indigenous responses were combined as Non-Hispanic Persons of Color in analyses.

#### Dismissiveness of Sexual Violence

The modified six-item version of the National Institute of Justice Survey of Attitudes and Behaviors Related to Sexual Harassment (Taylor et al., 2008) assessed student’s dismissive attitudes toward SV. Items included, “In my opinion… when boys make comments about girls' bodies, girls should take it as a compliment” and “In my opinion… sexual harassment is just having fun.” Response options ranged from 0 (*Strongly Disagree*) to 3 (*Strongly Agree*). Internal consistency for the scale was α = .85 (ω_h_ = .88).

#### Substance Use Intentions

Substance use intentions were assessed with four questions asking students how likely it would be that they would engage in substance use behaviors. Students were asked “How likely are you in the next 6 months to…”: (1) “smoke cigarettes,” (2) “get drunk or very high on alcohol,” (3) “use marijuana,” and (4) “use prescription drugs to get high.” Response options were: 0 (*Not at all likely*), 1 (*Somewhat likely*), and 2 (*Very likely*). As validity evidence, intent to use was associated with actual drug use in a longitudinal sample of adolescents (*n* = 847; Maddahian et al., 1988). Internal consistency for the scale was α = .76 (ω_h_ = .83).

#### Frienship Networks

 Students provided the first and last name of up to seven of their closest friends at their school. Ninox (2020), an online relational database software program, was used to search each school’s roster and match friends’ names with an anonymous student identification number. Of the 7489 nominations, 70.9% were successfully matched to school rosters. Friendship nominations were used to compute five measures with the sna (Butts, 2020) and igraph (Csardi & Nepusz, 2006) packages in R (R Core Team, 2020): (1) Indegree—the number of friendship nominations received—captures students’ popularity, (2) Outdegree—the number of friendship nominations made—captures students’ engagement in the school-based peer network, (3) Coreness captures students’ integration in the network, (4) Reciprocation rate captures relationship quality, and (5) Egocentric density captures the extent a friendship group is tightly connected. The second analysis added friend characteristic variables for students who named a friend (i.e., outdegree >0). These included variables addressing the heterogeneity of friendships (proportion of friends who were the same gender, race, and sexual orientation), the prevalence of SV within the friend group (proportion of friends who had previously (Waves 1–3) or concurrently (Wave 4) reported perpetrating SH or FSC), attitudes and behaviors of friends (friends’ average dismissiveness of SV and likelihood of substance use), and sports participation (proportion of friends who played no sports, non-, low-, or high-contact sports).

***Connections to Trusted Adults*** Students also nominated up to seven adults in their school who they would go to for help for themselves or for a peer (Wyman et al., 2019). In addition to the total number of trusted adults named, six dichotomous variables were created indicating whether the student had nominated a school administrator, teacher, counselor, health worker (e.g., nurse, social worker), paraprofessional, or support staff (e.g., bus driver, cafeteria worker).

### Data Analysis

All analyses were conducted in R (R Core Team, 2020). We addressed missing data by creating 50 imputed datasets via chained equations with the mice package (Van Buuren & Groothuis-Oudshoorn, 2011). Separate imputation models were built for the first and second analysis samples. The first analysis included the full sample of students. Descriptive statistics were calculated on the outcomes (SH and FSC perpetration in Wave 4) and other covariates by sport contact level. The bivariate association between sport contact level and each variable was examined via linear or logistic regression with *D*_*1*_ calculated as the pooled test statistic (Grund et al., 2016), and *p*-values adjusted for multiple comparisons with the Benjamini and Hochberg (1995) procedure. Perpetration in Wave 4 was then regressed on sport contact level, peer network, trusted adult, dismissiveness, substance use intent, a binary indicator for intervention group, and demographic variables using a single-level logistic regression model. Prior perpetration is highly predictive of later perpetration (Cutbush et al., 2016). Therefore, Model 1 excluded prior perpetration in order to directly address our research questions. Model 2 then added prior perpetration to illuminate how associations changed in the presence of a known predictor. Separate analyses were run for SH and FSC perpetration outcomes. The intraclass correlation was low for both outcomes (SH: ρ = .026; SV: ρ = .018). Nonetheless, cluster robust standard errors were calculated with the miceadds package (Robitzsch & Grund, 2021) to adjust for the clustering of students within schools.

The second analysis was intended to further explore the role of peer networks in SH and FSC perpetration by examining the association with friend characteristics. Therefore, the sample only included the 1665 students who named a friend. Descriptive statistics and the bivariate association between sports contact level and friendship characteristics were calculated. Two models—with and without prior perpetration—were then run regressing the perpetration outcomes on friendship characteristics and significant variables retained from the first analysis.

## Results

### Analysis 1 - Sports Contact Level, Peer Networks, and Trusted Adults

***Descriptive statistics*.** Differences in variables by sports contact level are presented in Table 1. The bivariate regression models indicated significant differences in both Wave 4 and prior SH perpetration rates by sports contact level. The low-contact group had the highest rates with 20.3% reporting SH perpetration in Wave 4 and 35.6% perpetrating in Waves 1–3. The no sports group had the lowest rates with 16.8% perpetrating in Wave 4 and 28.9% in prior waves. Differences in prior and Wave 4 FSC perpetration were non-significant. Significant differences by sports contact level were found across all social network indicators. Sports non-participants nominated fewer friends (outdegree), received fewer nominations (indegree), were less central in their networks (coreness), and had less connected networks (egocentric density) than sports participants. Non-participants also nominated fewer trusted adults and were less likely to nominate a school administrator, counselor or teacher as a trusted adult than sports participants. Thus, the descriptive statistics indicate sports participants across contact levels were more connected to both peers and adults in school than non-participants. Dismissiveness of SV differed significantly by sports contact level with the highest scores in the high-contact sports group and the lowest scores in the no sports group. Substance use intentions did not differ by sports contact level. Lastly, sports contact level groups differed in composition as suggested by the significant *D*_*1*_ statistic for gender, race, ethnicity, and sexual orientation.

**Table 1.** Count (%) or Mean (SD) by Sport Contact Level Pooled Across 50 Imputed Datasets.

Variable	No Sports*n* = 1310.1	Non-Contact*n* = 389.4	Low-Contact*n* = 533.8	High-Contact*n* = 1233.4	*D*_*1*_	*p*
Wave 4 SH perpetration	220.2 (16.8%)	71.8 (18.5%)	108.3 (20.3%)	225.8 (18.3%)	5.96 (3, 3270.7)	0.001
Wave 4 FSC perpetration	87.3 (6.7%)	34.2 (8.8%)	50.3 (9.4%)	98.2 (8.0%)	2.32 (3, 3167.8)	0.093
Prior SH perpetration	378.2 (28.9%)	126.2 (32.4%)	189.8 (35.6%)	431.9 (35.0%)	7.22 (3, 3039.9)	0.000
Prior FSC perpetration	128.9 (9.8%)	41.8 (10.7%)	79.4 (14.9%)	165.5 (13.4%)	1.55 (3, 3067.4)	0.207
Indegree	1.03 (1.21)	1.28 (1.40)	1.36 (1.52)	1.23 (1.47)	12.08 (3, 3425.0)	0.000
Outdegree	1.35 (1.89)	1.63 (2.04)	1.66 (2.04)	1.61 (2.12)	9.19 (3, 3424.9)	0.000
Coreness	0.45 (0.58)	0.60 (0.70)	0.60 (0.72)	0.55 (0.68)	18.05 (3, 3425.0)	0.000
Egocentric density	0.04 (0.12)	0.06 (0.14)	0.07 (0.17)	0.05 (0.14)	4.73 (3, 3425.0)	0.004
TA total nominations	1.32 (1.85)	1.58 (2.11)	1.64 (2.07)	1.68 (2.13)	12.57 (3, 3424.9)	0.000
TA - administrator	20.0 (1.5%)	12.1 (3.1%)	17.6 (3.3%)	43.5 (3.5%)	6.29 (3, 3424.4)	0.001
TA - counselor	60.1 (4.6%)	34.7 (8.9%)	30.7 (5.7%)	79.5 (6.4%)	5.77 (3, 3424.9)	0.001
TA - health worker	90.6 (6.9%)	15.0 (3.9%)	25.1 (4.7%)	64.4 (5.2%)	1.63 (3, 3424.9)	0.197
TA - paraprofessional	21.4 (1.6%)	7.7 (2.0%)	15.7 (2.9%)	16.8 (1.4%)	2.18 (3, 3424.7)	0.107
TA - support staff	66.7 (5.1%)	14.1 (3.6%)	35.9 (6.7%)	90.2 (7.3%)	2.00 (3, 3424.8)	0.129
TA - teacher	499.3 (38.1%)	162.1 (41.6%)	225.2 (42.2%)	526.0 (42.6%)	6.08 (3, 3424.9)	0.001
Substance use intent	0.24 (0.41)	0.24 (0.45)	0.30 (0.48)	0.26 (0.43)	1.40 (3, 3250.5)	0.240
SV dismissiveness	0.51 (0.52)	0.53 (0.60)	0.55 (0.60)	0.59 (0.57)	18.73 (3, 3275.6)	0.000
Girl	659.9 (50.4%)	180.9 (46.5%)	327.1 (61.3%)	452.2 (36.7%)	20.53 (3, 3423.8)	0.000
Other gender	35.4 (2.7%)	16.5 (4.2%)	15.0 (2.8%)	20.0 (1.6%)	2.56 (3, 3411.6)	0.072
Hispanic	652.0 (49.8%)	114.0 (29.3%)	212.8 (39.9%)	613.1 (49.7%)	22.98 (3, 3424.5)	0.000
Person of color	860.2 (65.7%)	170.4 (43.8%)	304.4 (57.0%)	801.4 (65.0%)	21.72 (3, 3424.5)	0.000
Sexual minority	334.5 (25.5%)	91.5 (23.5%)	142.6 (26.7%)	198.1 (16.1%)	15.03 (3, 3423.3)	0.000
Intervention	849.2 (64.8%)	241.9 (62.1%)	298.8 (56.0%)	679.7 (55.1%)	20.71 (3, 3425.0)	0.000

*Note.* SH = sexual harassment; FSC = forced sexual contact; SV = sexual violence; TA = trusted adult.

*D*_*1*_ is a pooled test statistic for effects in a regression model (Grund et al., 2016).

***Logistic regression***. The first research question examined whether perpetration rates differed by sport contact level and sport non-participation. Despite the bivariate association between sport contact level and SH perpetration, no association was found between sport contact level and SH or FSC perpetration when adjusting for peer network, trusted adult, and demographic variables in the logistic regression model, regardless of whether prior perpetration was excluded (Model 1) or included (Model 2) as a covariate (Table 2). The second research question investigated whether a FSC- or SH-sports link was moderated by gender, SV dismissiveness, or substance use intentions. For both the SH and FSC perpetration models, all of the interaction terms were non-significant, suggesting that no moderating effect was detected. As main effects, even when adjusting for prior perpetration (Model 2), intention to use substances was positively associated with SH perpetration (*OR* = 2.74; 95% CI: 1.88–4.00) and FSC perpetration (*OR* = 2.49; 95% CI: 1.28–4.87). Likewise, in Model 2, dismissiveness of SV was positively associated with SH (*OR* = 3.28; 95% CI: 2.14–5.03) and FSC perpetration (*OR* = 4.22; 95% CI: 2.25–7.91). The third research question investigated whether the number or type of trusted adults were associated with SH or FSC perpetration, but none of the trusted adult variables were statistically significant. Collectively, the Model 1 variables explained 18% and 23% of the variation in SH and FSC perpetration, respectively (Tjur, 2009). Prior perpetration explained an additional 5% and 3% of the variation, respectively.

**Table 2.** Pooled Odds Ratio (95% Confidence Interval) of Perpetration from Cluster Robust Logistic Regression Models for 3506 Adolescents.

Variable	Sexual Harassment		Forced Sexual Contact	
	Model 1	Model 2	Model 1	Model 2
Intercept	0.07 (0.03, 0.13)*	0.04 (0.02, 0.08)*	0.02 (0.00, 0.05)*	0.01 (0.00, 0.04)*
Non-contact	1.25 (0.44, 3.53)	1.26 (0.43, 3.69)	1.45 (0.27, 7.68)	1.44 (0.27, 7.79)
Low-contact	1.70 (0.66, 4.39)	1.60 (0.61, 4.17)	1.90 (0.38, 9.43)	1.69 (0.34, 8.34)
High-contact	1.08 (0.42, 2.80)	1.05 (0.39, 2.79)	1.30 (0.21, 8.00)	1.26 (0.18, 8.56)
Indegree	1.07 (1.00, 1.16)	1.09 (1.00, 1.19)*	0.92 (0.79, 1.06)	0.92 (0.79, 1.06)
Outdegree	0.97 (0.92, 1.03)	0.97 (0.92, 1.03)	0.92 (0.82, 1.04)	0.92 (0.80, 1.06)
Coreness	1.07 (0.86, 1.33)	1.00 (0.79, 1.26)	1.15 (0.76, 1.73)	1.12 (0.72, 1.73)
Egocentric density	1.01 (0.47, 2.15)	1.14 (0.52, 2.50)	0.73 (0.19, 2.85)	0.82 (0.22, 3.15)
TA total nominations	0.99 (0.90, 1.09)	0.98 (0.88, 1.08)	1.03 (0.92, 1.15)	1.02 (0.90, 1.15)
TA - administrator	1.09 (0.62, 1.92)	1.06 (0.58, 1.95)	1.27 (0.35, 4.63)	1.14 (0.28, 4.66)
TA - counselor	1.20 (0.76, 1.91)	1.23 (0.77, 1.96)	1.12 (0.55, 2.29)	1.21 (0.58, 2.50)
TA - health worker	0.70 (0.47, 1.05)	0.71 (0.47, 1.07)	0.46 (0.19, 1.12)	0.47 (0.19, 1.13)
TA - paraprofessional	0.64 (0.24, 1.71)	0.62 (0.21, 1.80)	0.37 (0.05, 2.99)	0.32 (0.04, 2.52)
TA - support staff	0.98 (0.72, 1.34)	1.07 (0.76, 1.52)	1.12 (0.51, 2.42)	1.12 (0.47, 2.69)
TA - teacher	1.22 (0.77, 1.93)	1.28 (0.80, 2.06)	0.97 (0.59, 1.59)	0.98 (0.57, 1.68)
Substance use intent	3.29 (2.29, 4.74)*	2.74 (1.88, 4.00)*	2.95 (1.52, 5.73)*	2.49 (1.28, 4.87)*
SV dismissiveness	3.49 (2.27, 5.36)*	3.28 (2.14, 5.03)*	4.53 (2.45, 8.38)*	4.22 (2.25, 7.91)*
Girls	0.49 (0.32, 0.77)*	0.57 (0.37, 0.89)*	0.63 (0.27, 1.50)	0.75 (0.31, 1.82)
Other gender	1.16 (0.62, 2.15)	1.37 (0.75, 2.53)	1.19 (0.56, 2.57)	1.33 (0.59, 2.99)
Hispanic	0.95 (0.72, 1.27)	1.04 (0.78, 1.37)	1.02 (0.57, 1.84)	1.15 (0.61, 2.19)
Person of color	0.99 (0.81, 1.22)	0.94 (0.76, 1.17)	1.09 (0.64, 1.86)	1.00 (0.55, 1.82)
Sexual minority	1.72 (1.33, 2.22)*	1.55 (1.22, 1.98)*	1.79 (1.30, 2.46)*	1.60 (1.18, 2.15)*
Intervention	0.97 (0.71, 1.33)	0.99 (0.74, 1.33)	0.94 (0.68, 1.28)	0.96 (0.69, 1.35)
High-contact x girls	1.23 (0.60, 2.53)	1.24 (0.58, 2.62)	1.25 (0.36, 4.33)	1.29 (0.34, 4.85)
Low-contact x girls	0.99 (0.41, 2.41)	0.98 (0.40, 2.43)	0.89 (0.21, 3.78)	0.89 (0.21, 3.82)
Non-contact x girls	1.03 (0.39, 2.72)	0.93 (0.35, 2.51)	1.15 (0.26, 5.17)	1.10 (0.22, 5.46)
High-contact x SVD	0.94 (0.47, 1.88)	0.90 (0.44, 1.84)	0.89 (0.31, 2.57)	0.84 (0.28, 2.46)
Low-contact x SVD	0.59 (0.31, 1.12)	0.58 (0.30, 1.10)	0.64 (0.26, 1.59)	0.65 (0.27, 1.60)
Non-contact x SVD	0.70 (0.31, 1.56)	0.73 (0.31, 1.72)	0.94 (0.38, 2.30)	1.00 (0.39, 2.55)
High-contact x SUI	0.71 (0.41, 1.22)	0.70 (0.39, 1.26)	0.92 (0.41, 2.06)	0.93 (0.41, 2.13)
Low-contact x SUI	1.04 (0.53, 2.01)	1.06 (0.53, 2.13)	1.29 (0.53, 3.15)	1.26 (0.50, 3.18)
Non-contact x SUI	1.12 (0.39, 3.20)	1.09 (0.42, 2.83)	0.52 (0.15, 1.80)	0.54 (0.14, 2.02)
Prior SH perpetration		3.34 (2.72, 4.10)*		1.68 (1.17, 2.40)*
Prior FSC perpetration		1.12 (0.84, 1.48)		3.09 (2.09, 4.56)*
Tjur’s *R*^2^	0.18 (0.15, 0.21)	0.23 (0.20, 0.25)	0.23 (0.19, 0.27)	0.26 (0.22, 0.29)

*Note.* SH = sexual harassment, FSC = forced sexual contact, SV = sexual violence, TA = trusted adult, SVD = sexual violence dismissiveness; SUI = dubstance use intent.

Respondents’ prior SH and FSC perpetration were excluded in Model 1 and included in Model 2. *p < .05.

### Analysis 2 - Friendship Characteristics

***Descriptive statistics***. Table 3 displays friendship characteristics disaggregated by sport contact level. There were significant differences in the percent of friends reporting prior SH and FSC perpetration. The high-contact group had the highest percentage with an average of 28.6% and 10.8% of their friends previously perpetrating SH and FSC, respectively. The high-contact group also had friends with the highest average SV dismissiveness score (*M* = 0.47, *SD* = 0.40) whereas the no sports group had friends with the lowest average SV dismissiveness score (*M* = 0.36, *SD* = 0.32). The percent of friends who were the same gender and the same sexual orientation differed significantly by sport contact level. The high-contact group reported the highest average percentage on both measures (same gender = 72.2%; same sexual orientation = 69.5%), which suggests greater homogeneity in their friendship network than the low-contact, non-contact, or no sports groups. Significant differences were also found for the percent of friends in each contact level along the expected trend (i.e., the non-contact group reported the highest percentage of friends participating in non-contact sports).

**Table 3.** Mean (SD) of Friend Characteristics by Sport Contact Level Pooled Across 50 Imputed Datasets.

Variable	No Sports*n* = 572.4	Non-Contact*n* = 192.6	Low-Contact*n* = 274.8	High-Contact*n* = 618.3	*D*_*1*_	*p*
Reciprocation rate	0.22 (0.30)	0.24 (0.30)	0.28 (0.33)	0.22 (0.28)	2.24 (3, 1648.0)	0.110
%Fr prior SH perpetration	23.54 (29.16)	26.85 (30.54)	26.07 (30.22)	28.61 (30.67)	3.51 (3, 1495.2)	0.026
%Fr prior FSC perpetration	7.55 (17.38)	7.51 (18.74)	9.63 (20.63)	10.76 (21.03)	3.00 (3, 1485.5)	0.046
%Fr W4 SH perpetration	12.94 (22.62)	14.34 (22.13)	13.99 (24.06)	15.78 (24.04)	2.20 (3, 1535.5)	0.110
%Fr W4 FSC perpetration	4.27 (14.04)	5.43 (15.11)	5.10 (15.65)	4.91 (14.09)	0.19 (3, 1482.2)	0.905
Avg. Fr SV dismissiveness	0.36 (0.32)	0.39 (0.32)	0.41 (0.39)	0.47 (0.40)	11.07 (3, 1439.2)	0.000
Avg. Fr substance use intent	0.18 (0.28)	0.20 (0.30)	0.20 (0.25)	0.21 (0.29)	1.98 (3, 1534.0)	0.134
%Fr same gender	67.08 (35.32)	66.95 (35.10)	69.29 (34.92)	72.15 (31.35)	5.55 (3, 1648.0)	0.002
%Fr same race	57.30 (40.37)	61.01 (39.88)	52.66 (39.89)	57.30 (40.35)	1.43 (3, 1648.0)	0.250
%Fr same sexual orientation	58.12 (39.16)	63.16 (38.06)	60.46 (39.27)	69.45 (37.05)	11.06 (3, 1648.0)	0.000
%Fr No sports	30.34 (30.95)	20.96 (28.08)	19.67 (29.23)	17.24 (27.83)	20.92 (3, 1241.7)	0.000
%Fr non-contact	6.23 (15.90)	16.99 (24.86)	7.51 (17.92)	5.52 (14.30)	17.57 (3, 1174.4)	0.000
%Fr low-contact	7.96 (17.65)	9.63 (19.41)	18.01 (25.90)	11.12 (20.88)	10.49 (3, 1271.9)	0.000
%Fr high-contact	18.76 (26.08)	22.14 (26.33)	25.20 (27.91)	37.51 (32.76)	40.59 (3, 1302.7)	0.000

*Note.* SH = sexual harassment; FSC = forced sexual contact; SV = sexual violence; Fr = friends; Avg = average.

*D*_*1*_ is a pooled test statistic for effects in a regression model (Grund et al., 2016).

***Logistic regression***. Although friendship characteristics differed by sport contact level, few were significantly associated with SH or FSC perpetration (Table 4). When excluding prior perpetration (Model 1) and adjusting for sports participation, the percent of friends who perpetrated FSC in Wave 4 was positively associated with FSC perpetration (*OR* = 1.02; 95% CI: 1.00–1.03) and the percent of same gender friends was negatively associated with FSC perpetration (*OR* = 0.99; 95% CI: 0.99–1.00). Percent of friends in a low-contact sport was positively associated with FSC perpetration (*OR* = 1.02; 95% CI: 1.00–1.03) and percent of friends in a high-contact sport was positively associated with SH perpetration (*OR* = 1.01; 95% CI: 1.00–1.01) even after adjusting for prior perpetration (Model 2). However, the effect size in each of these instances was extremely small.

**Table 4.** Pooled Odds Ratio (95% Confidence Interval) of Perpetration from Cluster Robust Logistic Regression Models For 1665 Adolescents Who Named A Friend.

Variable	Sexual Harassment		Forced Sexual Contact	
	Model 1	Model 2	Model 1	Model 2
Intercept	0.10 (0.05, 0.22)*	0.09 (0.04, 0.20)*	0.02 (0.01, 0.04)*	0.01 (0.01, 0.04)*
Non-contact	1.18 (0.59, 2.37)	1.12 (0.54, 2.29)	0.90 (0.26, 3.14)	0.81 (0.22, 2.92)
Low-contact	1.41 (0.76, 2.63)	1.25 (0.66, 2.39)	1.33 (0.46, 3.86)	1.08 (0.37, 3.18)
High-contact	1.04 (0.59, 1.82)	0.94 (0.53, 1.67)	1.31 (0.54, 3.18)	1.06 (0.43, 2.62)
Substance use	3.06 (2.22, 4.21)*	2.44 (1.75, 3.42)*	2.66 (1.67, 4.24)*	1.94 (1.12, 3.35)*
SV dismissiveness	3.05 (2.18, 4.27)*	2.49 (1.81, 3.44)*	4.09 (3.01, 5.54)*	3.09 (2.03, 4.69)*
Girl	0.49 (0.38, 0.64)*	0.54 (0.42, 0.69)*	0.71 (0.43, 1.17)	0.77 (0.46, 1.29)
Other gender	1.47 (0.59, 3.64)	1.56 (0.62, 3.94)	1.86 (0.55, 6.32)	1.86 (0.53, 6.57)
Sexual minority	1.51 (0.92, 2.48)	1.30 (0.81, 2.10)	1.01 (0.49, 2.10)	0.79 (0.41, 1.52)
Indegree	1.04 (0.89, 1.22)	1.03 (0.88, 1.22)	1.02 (0.85, 1.22)	1.02 (0.86, 1.22)
Reciprocation rate	1.05 (0.59, 1.88)	1.05 (0.58, 1.89)	0.49 (0.16, 1.49)	0.47 (0.16, 1.40)
%Fr prior SH perpetration	1.01 (1.00, 1.01)	1.00 (1.00, 1.01)	1.00 (1.00, 1.01)	1.01 (1.00, 1.01)
%Fr prior FSC perpetration	1.00 (0.99, 1.00)	1.00 (0.99, 1.01)	0.99 (0.97, 1.01)	0.99 (0.98, 1.01)
%Fr wave 4 SH perpetration	1.00 (1.00, 1.01)	1.00 (0.99, 1.01)	1.00 (0.99, 1.01)	1.00 (0.99, 1.01)
%Fr wave 4 FSC perpetration	1.01 (1.00, 1.02)	1.01 (0.99, 1.02)	1.02 (1.00, 1.03)*	1.02 (1.00, 1.03)
Avg. Fr SV dismissiveness	0.59 (0.31, 1.10)	0.59 (0.32, 1.10)	0.54 (0.23, 1.30)	0.50 (0.22, 1.15)
Avg. Fr substance use intent	0.64 (0.36, 1.13)	0.64 (0.34, 1.20)	0.67 (0.24, 1.85)	0.68 (0.21, 2.17)
%Fr same gender	1.00 (0.99, 1.00)	1.00 (0.99, 1.00)	0.99 (0.99, 1.00)*	0.99 (0.99, 1.00)
%Fr same race	1.00 (1.00, 1.00)	1.00 (1.00, 1.00)	1.00 (0.99, 1.00)	1.00 (0.99, 1.00)
%Fr same sexual orientation	1.00 (0.99, 1.00)	1.00 (0.99, 1.00)	1.00 (0.99, 1.01)	1.00 (0.99, 1.01)
%Fr No sports	1.00 (0.99, 1.01)	1.00 (0.99, 1.01)	1.01 (1.00, 1.02)	1.01 (0.99, 1.02)
%Fr non-contact	1.00 (0.99, 1.01)	1.00 (0.99, 1.01)	1.00 (0.98, 1.03)	1.01 (0.98, 1.03)
%Fr low-contact	1.01 (1.00, 1.02)	1.00 (0.99, 1.01)	1.02 (1.00, 1.03)*	1.02 (1.00, 1.03)*
%Fr high-contact	1.01 (1.00, 1.01)*	1.01 (1.00, 1.01)*	1.01 (0.99, 1.02)	1.01 (0.99, 1.02)
Prior SH perpetration		3.40 (2.70, 4.29)*		2.89 (1.63, 5.11)*
Prior FSC perpetration		1.19 (0.83, 1.69)		3.16 (1.76, 5.65)*
Tjur’s *R*^2^	0.15 (0.11, 0.19)	0.20 (0.16, 0.24)	0.12 (0.08, 0.16)	0.17 (0.13, 0.21)

*Note.* SH = sexual harassment; FSC = forced sexual contact; SV = sexual violence; Fr = friends; Avg = average.

Respondents’ prior SH and FSC perpetration were excluded in Model 1 and included in Model 2. *p < .05.

## Discussion

Collectively, the results showed a bivariate association where sports participants had higher SH perpetration rates than non-participants and were also more socially connected to peers and trusted adults than non-participants. Compared to non-participants and other contact levels, high-contact sports participants had the highest percentage of friend ties to prior SH and FSC perpetrators, friends with the highest average dismissiveness of SV, and the most demographically homogeneous friendships. Yet, neither sport contact level nor social network indicators were associated with perpetration when adjusting for the other variables in the logistic regression models. Most notably, in addition to prior SH and FSC perpetration, adolescents’ own dismissiveness of SV, and intention to use substances were strongly associated with SH and FSC perpetration. Although these individual factors (SV dismissiveness and intentions to use substances) were the strongest predictors of SH and FSC perpetration, some interesting findings related to sports and peers are worthy of further discussion.

First, no significant interactions were found between substance use intentions or SV dismissiveness with contact level of sport predicting SH or FSC perpetration. Descriptive findings, however, showed high- and low-contact sports participants were higher on intended substance use and SV dismissiveness than students in non-contact sports. This suggests that while SH and FSC perpetration is associated with substance use and dismissiveness rather than sports involvement in these data, sports may be a vehicle to develop, shape, and reinforce attitudes and behaviors that best predict SV perpetration. More nuanced research (e.g., qualitative analysis) is needed to fully understand sports participation as a potential contextual factor related to changes in perpetration (e.g., attributes of sports, role of coaches). Further, the construct of sport contact level in research might require refinement or augmentation with other aspects of sports. The current construct seems to result in classifying boys into high- and girls into low-contact sports and may not adequately capture the norms in certain sports undergirding SV perpetration (e.g., homophobic language). Nonetheless, our results align with previous gender attitude research for specific sports. For instance, McCauley and colleagues (2014) found in a sample of adolescent boys that, compared to all athletes, those who played the high-contact sports of basketball and football had significantly higher gender-inequitable attitudes whereas those who played the non-contact sports of tennis and swimming held significantly less inequitable attitudes.

Second, the Analysis 2 findings offer partial support for the MPS/pro-abuse peer theory. Respondents’ own participation in contact sports was not associated with SH nor FSC; yet, having friends in the high- and low-contact sports was associated with SH and FSC, respectively (albeit with small effect sizes). This lends some evidence for the role of peer support for violence within the context of sports. In addition, the percent of friends who were also current SH and FSC perpetrators became significant when the respondent’s own prior perpetration was excluded from the model. Thus, although the respondent’s own perpetration history is a more potent predictor of current SH and FSC perpetration, friends’ perpetration behavior also plays a role in explaining perpetration, further supporting peer support theory. Finally, the social network characteristics derived from friendship nominations (e.g., coreness and density of networks) were not associated with SH nor FSC perpetration. Yet, sports participants had strong social networks, and friendship with low- and high-contact sports participants was associated with perpetration. This aligns with previous research showing that adolescent SV perpetrators tend to be socially connected and to have friends who also perpetrate (Ybarra & Thompson, 2018). Thus, peer support may also play a role in shaping norms on sports teams that could lead to SV perpetration.

Findings reinforce the importance of sports in prevention, especially as it relates to promoting community social norms protecting against violence and teaching skills to prevent SV. The higher nominations of trusted adults among athletes in this study further reinforces the importance of utilizing coaches and other trusted adults as role models who can help shape prosocial and anti-violence norms among student-athletes. For example, the *Coaching Boys into Men* program, which provides coaches with resources to model and encourage respectful, non-violent, and healthy relationships for male athletes, has been found to effectively reduce dating violence, including SV perpetration, in both middle and high school (Miller et al., 2013; 2020). A recent review by Pringle and colleagues (2018) further indicates that the presence of trusted adults is associated with numerous positive outcomes among adolescents, including improved mental health and fewer risk behaviors (e.g., substance use). Sports medicine professionals can play an important role in this regard by adhering to SV prevention guidelines and providing services to athletes affected by SV (Joy et al., 2021). In addition, programs designed to teach and promote individuals’ social-emotional learning skills and healthy sexuality, while also addressing the physical environments of schools and communities that make SV perpetration more likely, are important for preventing SV in adolescence (Basile et al., 2016). These different efforts, coupled together, can create a comprehensive prevention approach that intervenes at individual, relationship, and community levels. Additionally, this study provides further support that SV prevention efforts would benefit from incorporating substance use prevention (Espelage et al., 2018), given it was an important predictor of SH and FSC perpetration. Finally, the importance of prior perpetration on later perpetration, found in this study and others, indicates that prevention efforts would benefit from beginning before high school (Espelage et al., 2015, 2018). Future research is needed to better understand friendship characteristics and social networks in middle school and how they may influence early substance use, dismissive of SV, and SV perpetration.

Subsequent research can also address the limitations of the present study. Most notably, although previous SH and FSC perpetration was taken into account, the associations between sports and perpetration were cross-sectional. Consequently, we cannot infer any causal conclusions. Future studies are needed to clarify the causal direction between sports participation and SH or FSC perpetration, should one exist. Another limitation of the present study was the lack of variability in perpetration reporting, which required us to reduce a multi-item scale to dichotomous indicators to fit the outcome models. The reduction in information may have yielded weaker and less nuanced associations between SH and FSC perpetration and the other variables. In addition, our measure of sports participation only included school sports; it is possible that some students participated in sports outside of school which was not captured. Similarly, the role of trusted adults was constrained to adults at school. Youth may have had close relationships with family, coaches, or other adults outside of school that impacted their propensity to perpetrate SH and FSC. Lastly, while the exploration of network and friendship characteristics is a unique contribution of the present study, over half of the sample (52.5%) did not nominate a friend. This might be another instance of underreporting, which further limits our ability to detect meaningful associations with SH and FCS perpetration and may decrease generalizability of our findings. Likewise, although the sample was representative of high school students in Colorado, there was a low proportion of non-Hispanic Persons of Color; thus, the findings may not extend to the broader national population.

This study enhances previous research by shedding light on the role and nature of adolescent social networks with both peers and adults in understanding the association of school sports participation with SV perpetration. Results indicate dismissive attitudes towards SV and intention to use substances, along with previous perpetration, have the strongest association with SV perpetration. Though our study did not find an association between sports participation and SV when adjusting for these other individual attitudes and behaviors, the extant literature continues to document cultures of interpersonal violence among sports teams (Young et al., 2017). The incongruence between our findings and this literature suggests that sports may be a vessel for the cultivation and reinforcement of attitudes and behaviors, including violent and supportive ones (Spruit et al., 2019). Our finding that sports participants were more connected to peers and trusted adults than non-participations also suggests that sports teams may have some influence on shaping both positive and negative norms. Further social network studies would be beneficial in this area, particularly analyses focused on friend groups as the unit of analysis to examine differences in SV perpetration, and a more nuanced understanding of the role of trusted adults, specifically coaches. Overall, findings suggest the sports context can be an important venue for prevention efforts by utilizing friendship networks and involving trusted adults to change norms around substance use, dismissive attitudes toward SV, and SV perpetration.
